# The Interplay Between Sleep Disorders and Cardiovascular Diseases: A Systematic Review

**DOI:** 10.7759/cureus.45898

**Published:** 2023-09-25

**Authors:** Rakshana Ravichandran, Lovish Gupta, Mansi Singh, Aiswarya Nag, Jingle Thomas, Binay K Panjiyar

**Affiliations:** 1 Department of Internal Medicine, Rajarajeswari Medical College and Hospital, Bangalore, IND; 2 Department of Internal Medicine, Maulana Azad Medical College, New Delhi, IND; 3 Department of Medicine, O. O. Bogomolets National Medical University, Kyiv, UKR; 4 Department of Internal Medicine, Sri Ramachandra Institute of Higher Education and Research, Chennai, IND; 5 Department of Internal Medicine, Al-Ameen Medical College, Vijayapura, IND; 6 Global Clinical Scholars Research Training (GCSRT) and Postgraduate Medical Education (PGME), Harvard Medical School, Boston, USA; 7 Department of Internal Medicine, California Institute of Behavioral Neurosciences and Psychology, Fairfield, USA

**Keywords:** heart failure, cardiac death, acute coronary syndrome, narcolepsy, insomnia, obstructive sleep apnea, hypertension, sleep apnea, cardiovascular disease, sleep disorders

## Abstract

Non-communicable diseases (NCDs) have emerged as the predominant cause of global mortality, resulting in a substantial annual loss of human lives. Among these conditions, cardiovascular diseases (CVD) stand out as the primary cause of death. The majority of CVD cases can be attributed to certain factors that, upon modification, have the potential to significantly decrease both the incidence and severity of the disease. For numerous years, the impact of sleep disorders on cardiovascular health has been a prominent subject of extensive discussion. Chronic sleep disturbances are known to have a range of negative health consequences, with the relationship between sleep apnea and hypertension being well-established through numerous studies. However, further exploration is needed to understand other disease associations with sleep apnea and to examine the impact of various sleep disorders, aside from sleep apnea, on cardiovascular health. This systematic review assesses the available evidence on the association between various sleep disorders and cardiovascular diseases by addressing the question: Do sleep disorders contribute to or exacerbate cardiovascular diseases? After a comprehensive review, we identified 122 articles. Following this initial review, seven papers directly aligned with our research topic. Subsequently, we meticulously assessed the remaining seven papers, all meeting our predetermined criteria. Our analysis showed a strong correlation between sleep disruptions and cardiovascular health. Numerous sleep disorders, such as narcolepsy, central sleep apnea, obstructive sleep apnea, and insomnia, have shown different effects on cardiovascular outcomes. Increased risks of illnesses such as acute coronary syndrome (ACS), hypertension, cardiovascular mortality, and coronary artery calcification were included in these consequences. This systematic review underscores the need for early identification and comprehensive management of sleep disturbances to mitigate their potential adverse effects on cardiovascular well-being. Integrating strategies that address sleep disorders and cardiovascular health is imperative in enhancing overall health outcomes. This study paves the way for more effective preventive and therapeutic approaches by focusing on the relationship between sleep disorders and cardiovascular diseases.

## Introduction and background

Over the past few decades, global health scenarios have transitioned from a primary focus on communicable diseases to an urgent concern for non-communicable diseases (NCDs). NCDs have emerged as the predominant cause of mortality, affecting 41 million people yearly, equivalent to 71% of all deaths globally. Of all NCD-related deaths, cardiovascular diseases (CVD) comprise the most significant portion, impacting 17.9 million individuals each year, constituting approximately 43% of NCD-associated mortalities [1]. One person dies every 33 seconds in the United States from CVD [2]. CVD is a collective term designating all types of afflictions affecting the blood circulatory system, including the heart and vasculature, which, respectively, displace and convey the blood [3]. Common diseases in this category include coronary artery disease (CAD), stroke, heart failure, arrhythmias, hypertension, and peripheral arterial disease (PAD). Risk factors for heart disease can be classified as either modifiable or non-modifiable. Modifiable risk factors account for over 70% of CVD cases and related deaths [4]. Although CVD encompasses a wide range of diseases, there are common behavioral factors that significantly increase the risk of developing these conditions. Therefore, addressing these shared risk factors will not only prevent the onset of CVD but can also aid in managing existing conditions and improving overall health. Sleep disorders comprise a group of conditions that disrupt normal sleep patterns. Patients with sleep disorders can be categorized into three groups: those who have trouble falling asleep (insomnia), those who experience behavioral and movement disturbances during sleep (rapid eye movement sleep behavior and restless legs syndrome), and those who suffer from excessive daytime sleepiness (narcolepsy) [5]. Sleep apnea is also characterized by interruptions in breathing during sleep, which results in frequent awakenings and disrupted sleep patterns. Approximately 40 million Americans experience chronic sleep and wakefulness disorders, impacting work, driving, and social activities [6]. These chronic sleep disturbances are associated with a range of adverse health consequences, including hypertension, CVDs, stroke, obesity, diabetes, migraines, and increased mortality [7,8]. Therefore, understanding the potential impact of sleep disorders on other health conditions is crucial to mitigate their adverse effects and promote overall well-being. The relationship between sleep apnea and hypertension has been extensively studied and well-established. However, further exploration is needed regarding other CVD associations with sleep disorders. Expanding our understanding of these associations is crucial for developing effective strategies to manage and prevent CVDs related to sleep disturbances. This systematic review assesses the available evidence on the association between various sleep disorders and CVD by addressing the question: Do sleep disorders contribute to or exacerbate CVDs?

## Review

Methods

**Figure 1:**
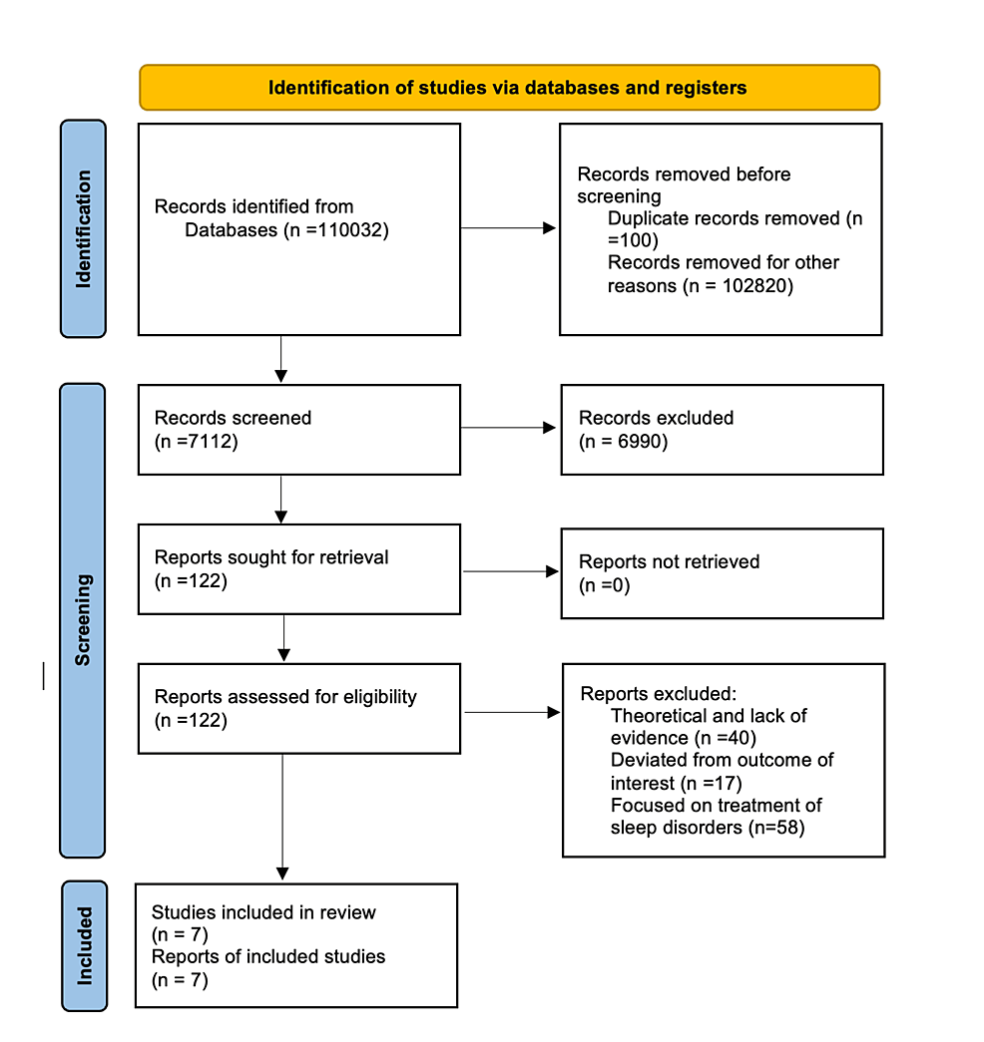
PRISMA flowchart demonstrating the search strategy and selection process of studies for the systematic review. PRISMA: Preferred Reporting Items for Systematic Reviews and Meta-Analyses.

Clinical investigations that have explored the association between sleep disturbances and CVD are the primary focus of this review. Animal studies and articles that merely mentioned sleep disturbances without demonstrating any link to cardiovascular ailments were eliminated. The review complies with the Preferred Reporting Items for Systematic Reviews and Meta-Analyses (PRISMA) for 2020 guidelines, as described in Figure 1 [9]. It relies solely upon data obtained from peer-reviewed publications, eliminating the need for ethical review.

Systematic Literature Search and Study Selection

We conducted an extensive review of the two widely utilized databases, namely PubMed and Google Scholar, to identify pertinent literature. We searched for studies referenced in randomized controlled trials, systematic reviews, clinical trials, and meta-analyses on PubMed. Subsequently, we independently assessed a collection of abstracts against pre-established criteria for inclusion. These criteria were centered on sleep disorders with connections to CVDs. Notably, we excluded studies involving animals. Four reviewers evaluated each abstract, and any differences in assessment were resolved through discussion.

Inclusion and Exclusion Criteria

To meet the objectives of our study, we developed precise standards for the inclusion and exclusion of individuals. Following is a list of our criteria presented in Table 1.

**Table 1:** Criteria for the inclusion and exclusion criteria applied during the literature search.

Inclusion Criteria	Exclusion Criteria
a) Human studies	a) Animal studies
b) From 2013- 2023	b) Only methodological studies explaining Programming details
c) English text	c) Non-English texts
d) Gender: All	d) Age <19 years of age
e) Age ≥19 years of age	e) Studies involving clinical data other than sleep disorders
f) Free papers	
g)Type of articles : systematic review, meta-analysis, clinical trials, randomized control trial	

Search Strategy

A systematic search was performed on databases, including PubMed and Google Scholar Libraries, using relevant keywords. PubMed utilized the Medical Subject Heading (MeSH) approach for study selection, followed by an initial screening based on titles and abstracts. Keywords such as 'sleep disorder' and 'cardiovascular diseases' were employed for the Google Scholar search. After reviewing the initial 18 pages of search results, a total of 66 potentially relevant articles were identified for further screening. The search strategies of the databases are summarized in Table 2.

**Table 2:** Search methodology, utilized search engines, and the count of retrieved results. MeSH: Medical Subject Heading.

Database	Search Strategy	Search results
Pubmed	((((((((sleep disorder[Title/Abstract]) OR (sleep apnea[Title/Abstract])) OR (sleep problems[Title/Abstract])) OR (breathing, sleep disordered[MeSH Terms])) OR (chronic insomnia[MeSH Terms])) OR (narcolepsy[MeSH Terms])) OR (abnormalities, heart[MeSH Terms])) OR (cardiovascular abnormalities[MeSH Terms])) OR (coronary disease[MeSH Terms])	7028
Google Scholar	(sleep disorders) AND (cardiovascular diseases)	103000

Quality Appraisal

Various quality assessment tools were used to ensure the reliability of the selected papers, as shown in Table 3. We used various quality assessment tools to ensure the validity of the seven papers we selected. For the systematic reviews and meta-analyses, we used the PRISMA checklist. Cochrane bias tool assessment was used for randomized clinical trials. The Newcastle-Ottawa tool scale was utilized to evaluate clinical studies that were not randomized. Using the Critical Appraisal Skills Program (CASP) checklist, we assessed the quality of the qualitative investigations. We used the scale to evaluate narrative review articles (SANRA) to rate the article's quality to prevent any ambiguity in the classification.

**Table 3:** Quality appraisal tools used. RCT: Randomized controlled trials; PRISMA: Preferred Reporting Items for Systematic Reviews and Meta-Analyses; SANRA: Scale for the Assessment of Narrative Review Articles.

Quality Appraisal Tools Used	Type of Studies
Cochrane Bias Tool Assessment	Randomized Control Trials
Newcastle-Ottawa Tool	Non-RCT and observational studies
PRISMA Checklist	Systematic reviews
SANRA Checklist	Any other without clear section

Results

We found 7032 papers in PubMed and 103000 articles in Google Scholar during the initial search employing keywords and Mesh terms, which yielded 110032 studies. We removed 100 duplicate entries, and after applying specific criteria, an additional 102,820 entries were excluded. After further title and abstract screening, we eliminated 6990 papers, leaving us with 122 articles. Seven papers that addressed our research topic were left after a thorough review. The next step was a complete quality review of the remaining seven papers that satisfied our standards. Our final systematic review includes these seven articles, as described in Table 4.

**Table 4:** Summary of the results of the selected papers. ACS: Acute coronary syndrome; OSA: Obstructive sleep apnea; CAC: Coronary artery calcification; AF: Atrial fibrillation; HF: Heart failure.

Author/Year	Country	Study Design	Database	Conclusion
Chung WS et al. (2013) [10]	Taiwan	Cohort study	National Health Insurance Research Database (NHIRD) in Taiwan	Longitudinal nationwide population-based cohort design and included 49,099 cases of non apnea sleep disorders and 98,198 control participants without sleep disorders showed non apnea sleep disorder cohort had a 1.43-fold higher risk of subsequent ACS compared to the cohort.
Sofi F et al. (2020) [11]	USA, Netherlands, Sweden, UK, Germany, Norway, France, Finland, Taiwan, Japan	Meta-analysis	MedLine, Embase, Web of Science, The Cochrane Library, Google Scholar, Clinicaltrials.org	13 prospective studies which showed insomnia is associated with a 45% increased risk of developing or dying from cardiovascular disease during follow-up.
Heilbrunn ES et al. (2021) [12]	North America, Europe, Asia, South America, Australia	Systematic Review and Meta-analysis	MedLine, Cochrane Library, SCOPUS and Joanna Briggs Institute	22 observational studies with a total of 42,099 participants estimated that individuals with OSA had a nearly twofold higher risk of cardiovascular mortality than those without OSA.
Hao W et al. (2021) [13]	Korea, Hungary, USA, Japan, Brazil, Turkey, Germany	Systematic Review and Meta-analysis	MedLine, Cochrane, and Google Scholar	OSA had a higher rate of Coronary Artery Calcification (CAC) presence and mean CAC scores compared to those without OSA.
Tung P et al. (2017) [14]	USA	Cohort study	Sleep Heart Health Study	2912 individuals were followed for an average of 5.3 years and found that Central Sleep Apnea was associated with incident AF, even after adjusting for cardiovascular risk factors.
Jennum PJ et al. (2021) [15]	Denmark, Italy, USA, France	Literature review	PubMed	Myocardial infarction, cardiac arrest, and heart failure were all significantly increased in people with narcolepsy versus controls.
Kanno Y et al. (2016) [16]	Japan	Case Control Study	Symptomatic HF patients (n=1,083) who were hospitalized for decompensated HF and were discharged from Fukushima Medical University between 2009 and 2013 were enrolled into this study.	Cardiac death and worsening HF was more among the insomnia group than non insomnia group.

Discussion

The intricate connection between sleep disturbances and cardiovascular health has recently gained increased attention from the medical and scientific communities. The pivotal role of sleep in maintaining overall health, including cardiovascular function, cannot be overstated. Various adverse cardiovascular outcomes, ranging from acute events like ACS to chronic conditions like heart failure, have been linked to disrupted sleep patterns. Obstructive sleep apnea (OSA) is characterized by periodic narrowing and obstruction of the pharyngeal airway during sleep [17]. The prevalence of hypertension in OSA patients is between 30 and 70% [18]. According to Phillips CL et al. study, sympathetic activation is a major mechanism through which OSA causes hypertension [19]. Besides hypertension, OSA plays a significant risk factor in other CVDs. The studies by Heilbrunn ES et al. [12] and Hao W et al. [13] are particularly notable for highlighting the impact of OSA on an elevated risk of cardiovascular mortality, as well as coronary artery calcification (CAC). This is in accordance with the 2005 retrospective study, which stated that the relative risk of sudden cardiac death (SCD) was 2.57-fold higher between midnight and 6 a.m. in patients with OSA compared with the general population. It also stated that this relative risk of SCD increased in proportion to the increasing severity of the apnea-hypopnea index (AHI), which is used to grade the severity of OSA [20]. In addition to OSA, central sleep apnea (CSA) has also emerged as a significant contributor to cardiovascular risk factors. CSA is characterized by a lack of drive to breathe during sleep, resulting in insufficient or absent ventilation and compromised gas exchange. In contrast to OSA, in which ongoing respiratory efforts are observed, CSA is defined by a lack of respiratory effort during cessations of airflow [21]. The study conducted by Tung P et al. states that despite adjusting for cardiovascular risk factors, CSA was associated with atrial fibrillation (AF) [14]. Müller M et al., in their study, show that heart failure patients without central sleep apnea syndrome (CSAS) experienced approximately twice the lifespan compared to those with CSAS [22].

Acute coronary syndrome (ACS) refers to conditions that experience a rapid decrease in coronary blood supply. There is a significant association between major cardiovascular risk factors, such as smoking, dyslipidemia, hypertension, and diabetes, with ACS, particularly in younger individuals [23]. In addition to conventional risk factors, sleep disturbances are emerging as a compelling and distinct contributor to the prevalence and progression of ACS. In a meta-analysis by Le Grande MR et al. following hospitalization for ACS, the combined occurrence rates were 22% for severe OSA and 70% for mild OSA [24]. Beyond sleep apnea, various other sleep disorders can potentially contribute as risk factors for ACS. This is supported by research conducted by Chung WS et al., indicating that individuals with non-apnea sleep disorders face an increased risk of ACS compared to those without sleep disorders [10]. Insomnia is a sleep disorder characterized by persistent difficulties in falling asleep, staying asleep, or returning to sleep after awakening. From a physiological standpoint, insomnia is linked to an elevated risk of adverse cardiovascular outcomes. A meta-analysis by Li L et al. demonstrates a significant association between insomnia and an increased risk of hypertension [25]. A study by Sofi F et al. reveals that insomnia is correlated with a 45% increased risk of developing or dying from CVD [11]. Insomnia can not only develop but also exacerbate existing conditions; as indicated by Kanno Y et al., heart failure symptoms worsen in individuals with insomnia compared to those without it [16].

Narcolepsy is a relatively common neurological sleep disorder, with a prevalence of 1 per 2000 people [26]. It exists in two distinct forms: Type 1 and Type 2. The more prevalent, Narcolepsy Type 1 (NT1), is characterized by excessive daytime sleepiness and episodes of uncontrollable sleep, during which individuals may appear immobilized and may even experience hallucinations. NT1 is often accompanied by cataplexy, a sudden loss of muscle tone or control leading to temporary muscle weakness or paralysis triggered by strong emotions. Conversely, Narcolepsy Type 2 (NT2) presents a more challenging array of symptoms and is not associated with cataplexy. Jennum P et al. review shows the risk for stroke was 2.5 times higher, heart failure 2.6 times higher, and all-cause death 1.5 times higher among individuals diagnosed with narcolepsy [15]. The Cardiovascular Burden of Narcolepsy Disease (CV-BOND) study further confirmed and expanded upon existing literature by demonstrating significantly higher incidence rates of multiple cardiovascular events, including stroke, heart failure, ischemic stroke, Major Adverse Cardiovascular Events (MACE), grouped instances of stroke, atrial fibrillation, edema, and CVD, in people with narcolepsy compared to matched non-narcolepsy controls [27]. This comprehensive investigation elucidates the intricate connections between sleep disorders and cardiovascular health. Although our analysis sheds important light on the complex associations between sleep disorders and cardiovascular health, it is essential to acknowledge several limitations. There might be a few high-quality studies that were excluded due to the unavailability of open access to their findings. The diversity in methodology, participant demographics, and research designs among the studies included may introduce variability in the findings, potentially impacting the generalizability of the conclusions and warranting further research to establish clinical correlations.

## Conclusions

This comprehensive study has shed light on the various effects of distinct sleep disorders on cardiovascular outcomes. The findings highlight the critical importance of prompt and accurate diagnosis of sleep disorders as a vital step in preventing and mitigating their detrimental impact on cardiovascular health. The need of the hour is the integration of healthcare strategies that address both sleep problems and cardiovascular health. Understanding and addressing these connections will aid in the development of more effective preventive measures and management techniques, ultimately enhancing cardiovascular health and overall quality of life.
